# The 4th Educational Course of the European Burns Association (EBA)

**DOI:** 10.3390/ebj7020030

**Published:** 2026-05-14

**Authors:** Nadia Depetris, Alette E. E. de Jong, Clemens Schiestl, Fredrik Huss, Gregoire Bondu, Jill Meirte, Jyrki Vuola, Luís Cabral, Moustafa Elmasry, Raluca Tatar Bulea, Robert Zajíček, Stian Almeland, Yvonne Wilson

**Affiliations:** 1 Burn Center, Centro Traumatologico Ortopedico, Città Della Salute e Della Scienza, 10126 Turin, Italy; 2 Burn Center, Rode Kruis Ziekenhuis, 1942 LE Beverwijk, The Netherlands; 3 Department of Plastic, Hand Surgery and Burn Care, BG Klinikum Bergmannstrost, 06112 Halle, Germany; 4 Department of Surgical Sciences, Plastic Surgery, Uppsala University, 751 85 Uppsala, Sweden; 5 Burn Center, Department of Plastic and Maxillofacial Surgery, Uppsala University Hospital, 751 85 Uppsala, Sweden; 6 Infirmier Centre de Traitement des Brules, Hôpital Saint-Louis, 75010 Paris, France; 7 Department of Rehabilitation Sciences and Physiotherapy REVAKI-MOVANT, Faculty of Medicine and Health Sciences, University of Antwerp, 2610 Wilrijk, Belgium; 8 Oscare, Organization for Burns, Scar After-Care and Research, 2170 Antwerp, Belgium; 9 Helsinki Burn Centre, Department of Plastic Surgery, Jorvi Hospital, Helsinki University Hospital, University of Helsinki, Karvasmäentie 8, 02740 Espoo, Finland; 10 Department of Plastic Surgery and Burns Unit, Coimbra University Hospital Centre (CHUC), 3000-075 Coimbra, Portugal; 11 Department of Hand Surgery, Plastic Surgery and Burns, Linköping University, 581 83 Linkoping, Sweden; 12 Department of Plastic Reconstructive Surgery, Grigore Alexandrescu Clinical Emergency Hospital for Children, Carol Davila University of Medicine and Pharmacy, 020021 Bucharest, Romania; 13 Department of Plastic Reconstructive Surgery and Burns, Grigore Alexandrescu Clinical Emergency Hospital for Children, 010621 Bucharest, Romania; 14 Prague Burn Centre, University Hospital Královské M‹ Vinohrady, Srobárova 50, 10034 Prague, Czech Republic; 15 Burn Center, The Third Medical Faculty, Charles University and The Kralovske Vinohrady University Hospital, 10034 Prague, Czech Republic; 16 Department of Plastic, Hand and Reconstructive Surgery, Norwegian National Burn Center, Haukeland University Hospital, NO-5021 Bergen, Norway; 17 Department of Clinical Medicine, University of Bergen, NO-5021 Bergen, Norway; 18 Burns Centre, Birmingham Women’s and Children’s NHS Foundation Trust, Birmingham B4 6NH, UK

**Keywords:** burns, burn care, burn center

## Abstract

Abstracts of the plenary sessions, workshops, and poster presentations of the 4th EBA Educational Course in Bucharest, Romania, 15–16 May 2026.

## 1. Introduction

Burn mass casualty incidents represent one of the most demanding challenges in disaster medicine, combining clinical complexity with the rapid saturation of highly specialized resources. Even well-developed burn care systems can be quickly overwhelmed, underscoring the need not only for effective response and preparedness, but also for strong prevention strategies.

The European Burns Association (EBA) Educational Course held in Bucharest addressed this continuum, prevention, preparedness, and responses, bringing together a truly multidisciplinary faculty and audience, including surgeons, intensivists, nurses, rehabilitation specialists, and other healthcare professionals involved in burn care. In a region characterized by diverse healthcare systems and resources, reducing the impact of burn disasters requires coordinated action across specialties and levels of care, as well as strong collaboration between institutions and countries.

The course provided a comprehensive and highly interactive platform to explore key aspects of burn mass casualty management, including prevention strategies, disaster preparedness, acute clinical care, surgical management, aftercare, and long-term system resilience. Through a dynamic combination of lectures, workshops, panel discussions, world café sessions, and case-based discussions, participants were actively engaged in exchanging experiences, reflecting on real-world scenarios, and contributing to shared problem-solving.

Particular emphasis was placed on integrating multidisciplinary perspectives into disaster planning and response, strengthening institutional, regional, and national preparedness, and enhancing European cooperation to ensure effective response when local capacities are exceeded. The course also highlighted the human dimension of burn disasters, including decision-making under pressure and the need to protect both patients and healthcare professionals.

This report summarizes the key themes and insights from the course, reflecting on current practices, shared challenges, and future priorities in advancing prevention, preparedness, and response to burn mass casualty incidents across Europe.

## 2. Acknowledgments

Thanks are due to all the EBA Committees who worked together to organize this meeting. The assistance of all the staff members of Congress Care and the Editorial Office of the European Burns Journal in preparing this congress is gratefully recognized. All the industries and companies which supported this congress are also appreciatively acknowledged. Moreover, deep gratitude is extended to all the professionals, researchers, burn survivors’ associations, and family members who actively and incessantly work to improve burn care.

## 3. Plenary Sessions

### 3.1. Plenary Session 1: Introduction

Moderators: Gordana Georgieva, Fredrik Huss

Burn mass casualty incidents represent a uniquely challenging subset of disasters, characterized by high clinical complexity, resource intensity, and rapid system saturation. Across Europe, such events—ranging from industrial accidents and transportation disasters to terrorist attacks and large-scale fires—have repeatedly exposed vulnerabilities in healthcare systems, even in well-resourced settings. This session provides an overview of burn mass casualty incidents in Europe, highlighting epidemiology, recurring patterns, and lessons learned from recent events.

Unlike other mass casualty scenarios, burn disasters overwhelm systems more rapidly due to the need for specialized care, including intensive care unit capacity, surgical expertise, infection control, and long-term rehabilitation. Even a relatively small number of severe burn patients can exceed local and regional surge capacity, creating immediate bottlenecks in triage, transport, and definitive care. Furthermore, the multidisciplinary and prolonged nature of burn treatment amplifies the strain on healthcare systems over time.

Preparedness plays a critical role in mitigating these challenges. Effective response requires coordinated planning at institutional, regional, and national levels, including surge capacity strategies, inter-hospital transfer agreements, stockpiling of essential resources, and regular simulation-based training. Cross-border collaboration and integration into broader disaster management frameworks are also essential in the European context.

This session will explore how preparedness frameworks can enhance resilience, improve patient outcomes, and support coordinated responses to BMCIs. By examining past incidents and current strategies, we aim to identify key priorities for strengthening burn disaster preparedness and response across Europe.

#### 3.1.1. Friday 15 May 2026 09:00–09:30

Overview of burn mass casualty incidents in Europe

Speaker: Raluca Tatar

Burn mass casualty incidents present significant challenges to healthcare systems due to the need for rapid triage, specialized care, and coordinated resource allocation. All over the world, such incidents, ranging from industrial explosions and transportation accidents to large-scale fires, have raised significant difficulties for the emergency response system of every affected country.

This presentation explores the burn-related mass casualty incidents in Europe, focusing on their main causes, frequency and outcomes. Regarding the main occurrence patterns and trends, industrial accidents and urban fires, in a variety of contexts, remain leading causes of these overwhelming situations. Key challenges include surge capacity limitations and the availability of specialized burn units and trained burn teams. Lessons learned from past incidents emphasize the importance of disaster preparedness planning, cross-border collaboration, and standardized triage protocols.

#### 3.1.2. Friday 15 May 2026 09:30–10:00

Why burn disasters overwhelm systems faster than other mass casualty events

Speaker: Thomas Leclerc

Why do burn disasters overwhelm systems faster than other mass casualty events? Burn disasters can be distinguished from other mass casualty events through the following specific features.

In medium-to-high revenue countries, severe burns are a rarely occurring condition. Nonetheless, their medical and surgical management is highly resource intensive and mobilizes large multidisciplinary teams of highly specialized healthcare professionals in burn centers. Accordingly, the paucity of burn centers is the main bottleneck of crisis management in a burn disaster, whereas other mass casualty events typically meet their largest challenge on scene.

Provided appropriate specialized burn care can be delivered, the lethality of severe burns is low compared to other trauma, typically more delayed, and considerably reduced by relatively simple initial management measures. This suggests the possibility that remarkable outcomes in most patients could be achieved even in large-scale burn disasters, provided a stepwise organization and optimal triage measures are implemented, as exemplified by recent events.

Finally, the high hospital length of stay of burned patients and their heavy human and logistical resource requirements, both in acute burn centers and then in specialized rehabilitation centers, cause a major and lasting strain on these structures.

This presentation discusses the aforementioned points and analyzes how it influences organizational choices for burn disaster response.

#### 3.1.3. Friday 15 May 2026 10:00–10:30

Role of preparedness at institutional, regional, and national level

Speaker: Stian Almeland

### 3.2. Plenary Session 2: Operating Under Extreme Surge

Moderators: Sophia Papadopoulou, Thomas Leclerc

What happens when the number of critically burned patients exceeds every available resource—within hours? This session explores the reality of operating under extreme surge. Operating under extreme surge conditions represents one of the greatest challenges in burn care, where the sudden influx of critically injured patients rapidly exceeds available resources and disrupts standard care pathways. Burn mass casualty incidents require immediate adaptation of clinical decision-making, triage processes, and resource allocation under highly constrained circumstances.

This session will explore the realities of managing extreme surge scenarios through the analysis of selected European case studies. Drawing from experiences in Bucharest (2015), North Macedonia (2025), and Switzerland (2026), speakers will highlight key operational challenges, including system overload, coordination of care, inter-hospital transfers, and maintenance of care standards in crisis conditions.

Particular attention will be given to how teams adapt in real time, balancing ethical considerations with clinical priorities, and implementing crisis standards of care when conventional approaches are no longer feasible. Lessons learned from these events will provide valuable insights into resilience, leadership, and system flexibility.

By examining these real-world experiences, this session aims to identify practical strategies to improve preparedness and response when operating under extreme surge, ultimately supporting more effective and coordinated management of future burn disasters.

#### 3.2.1. Friday 15 May 2026 16:00–16:30

Bucharest 2025

Speaker: Sorin Parasca

The presentation will provide a briefing on the events from October 2015 from Colectiv Club, Bucharest, where a massive fire took place with a large number of victims. A total of 27 people died on site or during transportation to hospital. A total of 146 patients were admitted to several hospitals in Bucharest. During the following days, another 33 patients died. A total of 35 patients were transferred at some point during their recovery to burn centers abroad. Thorough statistics on the recovery of patients will be provided. Inhalation injury and its complications proved to be the most prevalent cause of death among the patients. NexoBrid was used for the first time in Romania on patients involved in this accident.

Conclusions about the reaction of the authorities, the limits of the provided care, the mistakes and also the efforts made by the medical personnel will be discussed. The consequences of this terrible accident will also be described.

#### 3.2.2. Friday 15 May 2026 16:30–17:00

North Macedonia 2025

Speaker: Gordana Georgieva

In March 2025, a catastrophic fire in Kočani, North Macedonia, resulted in a mass casualty incident involving more than 200 patients, predominantly young individuals. Of these, 129 sustained cutaneous burns and 80 presented with inhalation injuries, with 38 requiring intubation. The scale and severity of injuries rapidly overwhelmed national healthcare capacities, necessitating immediate activation of a coordinated, multi-level response.

Initial management was guided by Advanced Trauma Life Support (ATLS) principles, with rapid triage, airway stabilization, and early fluid resuscitation forming the cornerstone of care. Patients were distributed across multiple healthcare facilities based on injury severity and resource availability. Given the limited capacity for advanced burn care, 115 patients were transferred to specialized burn centers abroad through international collaboration and activation of the European Civil Protection Mechanism.

This experience highlighted the critical importance of structured triage systems, inter-hospital coordination, and early decision-making regarding patient transfer. The high incidence of inhalation injury underscored the need for prompt airway management and intensive care preparedness. Furthermore, the incident emphasized the value of international networks in managing large-scale burn disasters, particularly for smaller healthcare systems.

Beyond the acute phase, ongoing challenges include long-term rehabilitation, reconstructive needs, and psychosocial support for survivors. The Kočani fire serves as a significant case study in disaster preparedness, demonstrating how coordinated national and international efforts can optimize outcomes in mass burn incidents and providing important lessons for future emergency response planning.

#### 3.2.3. Friday 15 May 2026 17:00–17:30

Switzerland 2026

Speaker: Clemens Schiestl

Shortly after midnight on New Year’s Eve, a fire broke out in a basement bar in the Swiss Alps. There were 164 people in the bar at the time. Within minutes, 40 people had lost their lives and 116 had suffered burns. Thanks to a unique rescue operation involving emergency services, burn centers and other hospitals, the initial wave of patients was successfully managed. Over the following two days, all patients requiring specialist treatment were transferred to burn centers in France, Belgium, Germany and Italy.

This presentation will outline the exact response sequence in Switzerland, as well as details of the collaboration between the Emergency Response Coordination Centre of the European Union, the European burn centers and the Swiss National Disaster Committee (KATAMED).

### 3.3. Plenary Session 3: Learning from Different Settings

Moderators: Nadia Depetris, Panche Taskov

Burn care delivery varies significantly across different healthcare settings, particularly where resources are limited, systems are under sustained pressure, or infrastructures are fragile. These contexts present unique and complex challenges that impact every aspect of care, from acute management to long-term rehabilitation.

This session will explore how burn care is adapted and delivered across diverse and challenging environments. Drawing on international experience, speakers will address capacity limitations, workforce constraints, supply chain challenges, and the impact of prolonged system stress on care delivery and patient outcomes.

Particular attention will be given to strategies that enable continuity and quality of care despite these constraints, including task sharing, adaptation of clinical protocols, prioritization of interventions, and strengthening of local capacity. The session will also highlight the importance of resilience, innovation, and context-specific solutions in fragile and resource-constrained settings.

By sharing experiences from different healthcare environments, this session aims to provide practical insights and transferable lessons to support clinicians and systems in delivering effective burn care under challenging conditions worldwide.

#### 3.3.1. Saturday 16 May 2026 09:00–09:30

Burn Care Capacity in Resource-Constrained Settings

Speaker: Roger Alcock

Burns patients require an integrated emergency, critical, operative and rehabilitation pathway which can be challenging in well-resourced settings, but in fragile, conflict-affected, low-resource environments good outcomes are even more difficult to achieve. This session will explore some of the challenges and solutions.

#### 3.3.2. Saturday 16 May 2026 09:30–10:00

Burn Care Delivery Under Prolonged Health System Stress

Speaker: Roman Chornopyshchuk

Mass casualty incidents represent a global challenge characterized by the sudden arrival of large numbers of casualties with a variety of injuries, particularly burns. Such events are accompanied by a significant number of casualties, severe combined injuries (including inhalation injuries, blast injuries and multiple trauma), as well as a high mortality rate. Consequently, they place a serious strain not only on the healthcare system, but also on other sectors.

This problem remains particularly acute in resource-constrained countries, where there is also a shortage of specialized burn centers, insufficient numbers of specialist staff, a lack of modern medical consumption materials, and so on. At the same time, additional difficulties arise against the backdrop of active armed conflict. A striking example is the situation in Ukraine, where the healthcare system is forced to operate under constant strain and adapt to the challenges of wartime.

An effective response to burn mass casualty incidents requires clearly coordinated multidisciplinary work, including the optimization of medical triage, rapid stabilization of patients’ conditions, and their rational distribution among healthcare facilities. Staff training, logistical readiness and the existence of clear protocols for action in crisis situations also play a significant role.

In this regard, the search for accessible yet effective response principles is of great importance, particularly in the context of limited resources and armed conflicts. Strengthening international cooperation, developing educational programs, and the rational use of available resources are key to improving outcomes for the treatment of patients with burns in mass casualty incidents.

#### 3.3.3. Saturday 16 May 2026 10:00–10:30

Burn Care in Fragile Health Care Environments

Speaker: Tomo Potokar

### 3.4. Plenary Session 4: Beyond Survival: Aftercare in Burn Mass Casualties

#### Saturday 16 May 2026 13:00–14:00

World Café Session: Jill Meirte, Koen Maertens

Survival after a burn mass casualty incident is only the beginning of a long and complex journey. Patients often require prolonged rehabilitation, psychosocial support, and coordinated follow-up care, placing sustained demands on healthcare systems and communities.

This session will provide an overview of the key challenges in aftercare following burn mass casualty incidents, including continuity of care, long-term outcomes, and the integration of multidisciplinary services. It will highlight the gaps that frequently emerge once the acute phase has passed, both at institutional and system levels.

Particular attention will be given to the role of structured pathways, rehabilitation services, mental health support, and community reintegration in improving patient outcomes.

Through an interactive World Café format, participants will have the opportunity to exchange experiences, identify priorities, and explore practical solutions to strengthen aftercare and ensure that patients receive the support they need beyond survival.

### 3.5. Plenary Session 5: National and European Response to Burn Mass Casualty Incidents

#### Saturday 16 May 2026 14:00–15:30

Moderators: Jill Meirte, Robert Zajicek

Panel: Sorin Parasca, Gordana Georgieva, Clemens Schiestl, Calin Alexandru, Thomas Leclerc, Dan Mircea Enescu

Burn mass casualty incidents represent a critical test of coordination, capacity, and resilience across healthcare systems. While local response remains essential, the scale and complexity of these events frequently require rapid escalation to regional, national, and even international levels of collaboration.

This panel will explore how European countries respond to large-scale burn disasters, focusing on the integration between national systems and supranational support mechanisms. Panelists will share experiences from past incidents, highlighting the challenges of patient distribution, cross-border transfers, communication, and resource mobilization under extreme pressure.

Particular attention will be given to existing European frameworks, cooperative networks, and the operational realities of implementing them in crisis situations. Differences in system organization, logistical barriers, and opportunities for harmonization will be critically discussed.

By bringing together diverse perspectives from across Europe, this session aims to identify strengths, expose gaps, and outline future directions to enhance coordinated responses to burn mass casualty incidents at both national and European levels.

### 3.6. Plenary Session 6: Is It Possible to Prevent Burn Mass Casualties? From Lessons Learned to Prevention and Policy

Moderators: Raluca Bulea Tatar, Sophia Papadopoulou

Burn mass casualty incidents are often perceived as unpredictable and unavoidable. However, repeated tragedies across Europe demonstrate that many of these events share common, preventable causes. Incidents such as nightclub and indoor fire disasters have highlighted how specific risk factors—particularly the use of pyrotechnics in enclosed spaces, inadequate safety measures, and insufficient regulation—can lead to catastrophic outcomes within minutes.

This session will explore whether burn mass casualties can be prevented, moving beyond response to focus on risk reduction and policy action. Drawing on lessons learned from past European incidents, speakers will examine how failures in safety standards, risk assessment, and enforcement contribute to large-scale burn disasters.

Particular attention will be given to the role of legislation and public health policy in prevention. Strategies such as banning the indoor use of fireworks and pyrotechnics in public venues, strengthening fire safety regulations, and promoting safer alternatives will be discussed as key measures to reduce risk. The importance of coordinated action between policymakers, healthcare professionals, and public authorities will be emphasized.

By linking clinical experience with prevention science and policy advocacy, this session aims to highlight actionable steps to reduce the incidence and severity of BMCIs. Ultimately, it will underline a critical message: many burn disasters are not inevitable, and effective prevention can save lives.

#### Saturday 16 May 2026 16:00–16:30

Is it possible to prevent burn mass casualties? From lessons learned to prevention and policy

Speaker: Koen Maertens

Recent tragedies across Europe have shown how quickly fire disasters can escalate, often driven by recurring and avoidable risk factors such as indoor pyrotechnics, inadequate safety measures, and regulatory gaps. These events continue to claim lives—often young—and leave many more with devastating injuries.

This session shifts the focus from response to prevention. Drawing on lessons learned from past incidents, we will explore how policy, legislation, and public health interventions can reduce the risk of burn disasters.

From banning the indoor use of fireworks and pyrotechnics to strengthening fire safety standards and promoting safer alternatives, speakers will highlight concrete actions that can make a difference.

Can we act before the next disaster happens?

### 3.7. Plenary Session 7: The Future of Burn Care

#### Saturday 16 May 2026 16:30–17:30

Panel discussion: Nadia Depetris, Raluca Tatar Bulea, Yvonne Wilson

Ensuring the future of burn care depends on the ability to attract, engage, and retain the next generation of healthcare professionals. Despite its clinical relevance and impact, the field faces challenges in maintaining interest among young clinicians, as well as providing clear pathways for career development and long-term engagement.

This session will explore the key factors influencing the involvement of young professionals in burn care across Europe. It will address barriers to entry, training opportunities, mentorship, and the importance of creating supportive and inspiring professional environments.

Particular attention will be given to strategies that foster motivation, professional growth, and multidisciplinary collaboration, as well as initiatives that promote visibility and accessibility of the specialty.

This session aims to identify practical approaches to strengthen engagement and ensure a sustainable and dynamic future for burn care.

## 4. Workshops

### 4.1. Workshop: Dressings in Burn Care

Sigrid Brokke, Grégoire Bondu, Klaudia Kokkola, Gaelle Smith, Dominique Potokar, Lottie Armitage, Alette de Jong

Optimal wound management is a fundamental component of burn care; it directly influences healing outcomes, infection prevention, pain, and long-term sequelae. Selecting and applying appropriate dressings for burn patients remains complex and requires a personalized approach that takes into account the depth of the burn, the stage of healing, the patient’s characteristics, and available resources.

In this workshop, we will address the issue of wound care in the event of burn mass casualties into our specialized centers. Mass influxes are rare and unpredictable, but they present our institutions with a real challenge. Faced with the potential need to provide a massive volume of care within a limited timeframe, with logistical challenges in mind, we as healthcare professionals must reflect on these issues.

Through a combination of discussion and hands-on sessions, we will explore this topic, drawing on feedback from the field and various dressing techniques. The aim of this session is to raise awareness of this specific aspect of burn wound management, thereby helping to improve the quality of care in our burn units.

### 4.2. Workshop: Triage in Burn Mass Casualties

Thomas Leclerc, Stian Almeland

Effective triage is a critical component in the management of burn mass casualty incidents, where rapid decision-making is essential to optimize outcomes and ensure the best use of limited resources.

This workshop will provide an overview of triage principles specific to burn disasters, including assessment of burn severity, prioritization of care, and allocation of patients to appropriate levels of treatment. Participants will explore the differences between conventional triage and burn-specific approaches in mass casualty settings.

Particular attention will be given to the challenges of performing accurate and consistent triage under pressure, as well as the importance of standardized protocols and training in improving response efficiency.

Through practical examples and interactive discussion, this workshop aims to strengthen participants’ understanding of burn triage and enhance preparedness for real-world scenarios.

### 4.3. Workshop: Acute Surgical Management

Raluca Tatar Bulea, Robert Zajicek, Tom Potokar, Roger Alcock

Acute surgical management is a cornerstone of burn care, playing a critical role in reducing morbidity and improving survival in patients with severe injuries. Timely and appropriate surgical intervention is essential to control infection, promote wound healing, and optimize functional and esthetic outcomes.

This session will provide an overview of key principles in the acute surgical management of burn patients, including early excision, wound coverage strategies, and the prioritization of surgical interventions in complex cases. It will also address decision-making in resource-limited and high-pressure environments, such as during burn mass casualty incidents.

Particular attention will be given to multidisciplinary coordination, perioperative considerations, and the integration of surgical care within the broader treatment pathway.

This session aims to highlight best practices and current approaches to acute burn surgery, supporting clinicians in delivering effective and timely care.

### 4.4. Workshop: Clinical Cases Discussion

Athina Lavrentieva, Clemens Schiestl, Gordana Georgieva, Roman Chornopyshchuk, Sophia Papadopoulou

The discussion of clinical cases offers a valuable opportunity to bridge theory and practice, particularly in the complex settings of burn care and burn mass casualty incidents.

This session will present a selection of representative cases from both routine clinical practice and mass casualty scenarios, highlighting key challenges in assessment, triage, treatment, and multidisciplinary management. Through structured analysis, participants will explore different approaches and consider the factors influencing decision-making under both standard and high-pressure conditions.

Particular attention will be given to critical decision points, complications, and lessons learned, including how strategies may need to adapt when resources are limited and demand is high.

This session aims to promote interactive learning, encourage exchange of experience, and support continuous improvement in both everyday burn care and disaster preparedness.

## 5. Poster Presentations

### 5.1. P01 Variation in Pro- and Anti-Inflammatory Factors in Severe Burns

**Dr Mihai-Codrin Constantinescu** ^1,2^, Dan Cristian Moraru ^1,2^, Stefana Avadanei-Luca ^1,2^, Andra-Irina Bulgaru-Iliescu ^1,2^, Alexandru-Hristo Amarandei ^1,2^, and Mihaela Pertea ^1,2^

1. Faculty of Medicine, Grigore T. Popa University of Medicine and Pharmacy Iasi, 700115 Iași, Romania.
2. Department of Plastic Surgery and Reconstructive Microsurgery, “Sf. Spiridon” Emergency County Hospital, 700111 Iași, Romania.

**Aim:** To identify pro- and anti-inflammatory mediators consistently associated with adverse outcomes in severe burns and evaluate their potential as immunomodulatory targets.

**Methods**: A PRISMA 2020-compliant systematic review searched PubMed, Embase, Cochrane Library, Web of Science, and Scopus (2006–2024). Eligible studies reported quantitative measurements of circulating cytokines, acute-phase reactants, complement fragments, or hematologic indices correlated with clinical outcomes (sepsis, ventilator-associated pneumonia, organ dysfunction, or mortality) in severe burn patients. Of the 1883 records identified, 24 met the inclusion criteria and were synthesized narratively.

**Results**: Pro-inflammatory cytokines (IL-6, IL-8, IL-1β, MCP-1, TNF-α) rose within 24–72 h and tracked burn size and severity. Non-survivors demonstrated persistent elevations of IL-6, IL-8, G-CSF, and MCP-1 compared to survivors. IL-8 independently predicted ventilator-associated pneumonia and mortality in mechanically ventilated patients. Anti-inflammatory mediators (IL-10, G-CSF, IL-4) increased early but showed variable kinetics. A meta-analysis (n = 1837) confirmed admission NLR as an independent mortality predictor. Composite hematologic indices (NLPR, SII, LPR) combined with burn severity scores achieved an AUC of 0.994. Complement activation and neutrophil extracellular trap markers (CitH3, neutrophil elastase) correlated with injury severity.

**Conclusions**: The post-burn inflammatory response is a dynamic, unstable equilibrium rather than a linear cascade. Outcomes are shaped by both the initial cytokine magnitude and the persistence of dysregulated inflammation. Composite biomarker panels integrating cytokines, hematologic ratios, and innate immune markers outperform single thresholds in prognostication and may guide phase-specific immunomodulation to improve survival in severe burns.

**Keywords:** burn injury; cytokines; inflammatory biomarkers

### 5.2. P02 Management of Fire Victims with Inhalation Injury in a Burn Intensive Care Unit: A Case Series

Chrysavgi Giannaki ^1^, Evangelos Kaimakamis ^1^, Spyridon Synodinos-Kamilos ^1^, Anastasios Tsangaleas ^1^, Stavros Tsilingeridis ^2^, Sophia Papadopoulou ^2^, and **Athina Lavrentieva** ^1^

1. Papanikolaou Hospital, A-ICU, Burn ICU, Thessaloniki, Greece
2. Papanikolaou Hospital, Plastic Surgery Department, Burn ICU, Thessaloniki, Greece

**Background:** The management of victims of mass disasters presents unique challenges and is associated with high morbidity and mortality. This study aims to describe the clinical management of fire victims admitted to a Burn Intensive Care Unit (ICU).

**Materials and Methods:** This is a descriptive study evaluating the following parameters: severity of burn injury, severity of overall clinical condition, therapeutic interventions during ICU stay, and patient outcomes.

**Results:** Three patients were evaluated who were transferred directly to the Burn ICU following a fire accident, presenting with thermal injuries and inhalation injury.

The mean total body surface area (TBSA) burned was 16.5 ± 4%, the Abbreviated Burn Severity Index (ABSI) was 6.3 ± 1.2, and the APACHE and SOFA scores were 10.6 ± 3.5 and 4.3 ± 0.5, respectively. All patients had grade 3 inhalation injury confirmed by bronchoscopy. They were sedated and required mechanical ventilation.

Each patient underwent repeated bronchoscopies resulting in improvement of the oxygenation index (PaO_2_/FiO_2_) from 147.8 ± 120 mmHg to 243 ± 100 mmHg and LUS (Lung Ultrasound Score) from 9 ± 5 to 3 ± 1.7 to 4.6 ± 3. Inhaled therapies (heparine, bronchodilators) were administered in all cases, while one patient received hydroxocobalamin due to persistent hemodynamic instability and metabolic (lactic) acidosis.

Two patients developed septic complications, required tracheostomy, and had prolonged ICU stays of 30 and 31 days, respectively. The third patient was successfully extubated after a 7-day ICU stay. One patient developed acute kidney injury but did not require renal replacement therapy. All three patients were diagnosed with deep venous thrombosis, and one patient additionally developed a pulmonary embolism.

**Conclusions:** Patients exposed to fire in enclosed spaces with delayed evacuation frequently develop inhalation injuries associated with a complicated clinical course and significant morbidity. Repeated bronchoscopic interventions play a crucial role in secretion clearance and improvement of respiratory function.

### 5.3. P04 Antibiotic Resistance of Opportunistic Pathogens in Combat-Injured Burn Patients: Role of Cationic Antiseptics in Burn Mass Casualty Preparedness

**Oleksandr Nazarchuk** ^1^, Roman Chornopyschuk, Vadym Starodub, Vira Bebyk ^1^, Kateryna Ksenchyna ^1^, Natalia Bahniuk ^1^, and Dmytro Dmytriiev ^1^

1. National Pirogov Memorial Medical University, Vinnytsya, Ukraine

**Aim:** To evaluate trends in antibiotic resistance among clinical isolates of opportunistic pathogens obtained from combat burn patients and to assess the antimicrobial activity of modern antiseptics against these pathogens.

**Methods:** This study analyzed trends in antibiotic resistance among clinical isolates of opportunistic microorganisms collected from 326 patients with combat trauma undergoing treatment in the burn unit. The search for alternative antimicrobial agents against predominant pathogens has become particularly relevant. The predominant isolates were *Acinetobacter* spp. (n = 77), *P. aeruginosa* (n = 94), *K. pneumoniae* (n = 76), *S. aureus* (n = 62), and *Proteus mirabilis* (n = 17).

**Results:** There was a high heterogeneity in antibiotic susceptibility (0–100%), with frequent multidrug resistance, especially in *Acinetobacter* spp. and *P. aeruginosa*. Carbapenems and amikacin retained better activity, while aztreonam was ineffective. Importantly, even highly resistant ESKAPE strains remained highly susceptible to the cationic antiseptics, with decamethoxin demonstrating the strongest bacteriostatic and bactericidal effect (*p* ≤ 0.001). This supports the use of local antiseptic treatment to reduce bacterial load, disrupt biofilms, and enhance systemic antibiotic efficacy.

**Conclusions:** The most promising strategy for treating infectious complications in combat burns is targeted systemic antibiotic therapy guided by susceptibility testing combined with regular local cationic antiseptic treatment in resource-limited settings.

**Keywords:** antiseptics; antibiotic resistance; burns; combat trauma; ESKAPE; opportunistic pathogens

### 5.4. P07 Clinical Efficacy, Advantages, and Application Method of Enzyme Alginogel in the Management of Superficial and Deep Partial-Thickness Burns: The Experience of Niguarda Burn Center

Laura Mammino ^1^ and **Federico Pili** ^1^

1. Niguarda Hospital, Milan, Milano, Italy

**Introduction:** Partial-thickness burns present complex challenges, including bacterial colonization risk, exudate management, and potential hypertrophic scarring. Optimal wound bed preparation is essential to accelerate re-epithelialization and prevent burn conversion into deeper dermal layers.

**Methods:** Enzyme alginogel integrates a hydrated alginate matrix with a bioactive antimicrobial enzyme system (glucose oxidase and lactoperoxidase). It promotes continuous selective autolytic debridement and controls bacterial load without cytotoxic agents (e.g., silver), thereby preserving keratinocytes and fibroblasts. The protocol involves applying a uniform layer of gel directly onto the wound bed, followed by an appropriate secondary dressing, with changes every 96 h depending on saturation.

**Results:** Clinical evidence indicates that enzyme alginogel significantly optimizes healing times in partial-thickness burns. Superior patient tolerance and optimal tissue quality are observed upon resolution. Furthermore, its application is associated with a marked reduction in pain during dressing changes and improved long-term esthetic and functional scar outcomes.

**Conclusions:** Enzyme alginogel represents a preferred therapeutic solution for second-degree burns. The synergy between selective debridement, non-cytotoxic antimicrobial protection, and high biocompatibility promotes rapid healing while minimizing permanent scarring complications.

**Keywords:** enzyme alginogel; partial-thickness burns; wound bed preparation; autolytic debridement; Niguarda Burn Center

### 5.5. P08 the Stock of Human Allogeneic Skin Grafts in the View of a Mass Disaster

**Phd Wojciech Łabuś** ^1^, Artur Wielgórecki ^1^, and Karolina Ziółkowska ^1^

1. Stanisław Sakiel Burn Treatment Center, Siemianowice Śląskie, Poland

**Aim:** To achieve and manage the stock of human allogeneic skin grafts in case of a mass disaster that may involve large numbers of severely burned patients.

**Methods:** This study involved an analysis of selected mass disasters. From this, an estimate was made from a verified casualty profile of the necessary minimum stock of human allogeneic skin graft materials. A proposal has been made for the organizational, legal and systemic changes required to improve the situation in Polish transplantology, with particular emphasis on skin donation.

**Results:** A government program has been established to create a strategic bank of biological dressings. In order to achieve a strategic stock of human skin grafts, a tissue-collecting transplantation team was organized. The rights and obligations of the non-physician transplant team member should be extended. Proposals have been made for awareness campaigns (adverts, posters, etc.) and educational schemes (educational videos, lectures during transplant coordinator training, etc.). Finally, a proposal has been made for possible methods to deal with the logistic management of the allogeneic skin stock.

**Conclusions:** The required, essential stock of human allogeneic skin in the event of a mass disaster has been estimated at 600,000 cm^2^.

### 5.6. P09 Advancing Civil and Military Burn Care by Integrating AI, Data Registration, and Education

**Dr. Patrick Mulder** ^1,2^, Dr. Barbara Verbeek ^1,2^, Col. Henk van der Wal ^1^, and Dr. Kees van der Vlies ^2^

1. CETC (UMC Utrecht and Ministry of Defence), Utrecht, The Netherlands
2. Burn Center, Maasstad Hospital, Rotterdam, The Netherlands

**Introduction/objectives:** Burn injuries in military settings pose a distinct clinical challenge, often affecting the hands and face. Inappropriate self-aid and buddy care, resource-limited settings and delayed access to specialized care increase the risk of infection, immune dysregulation, and long-term disability. We present a multidisciplinary approach on burn care aimed at achieving zero preventable mortality and minimizing disability after military burn trauma.

**Methods**: We will establish the prospective registration of military burn injuries, including evacuated patients (e.g., from Ukraine to the Netherlands). In parallel, artificial intelligence (AI) tools, including a decision-support mobile application, will be developed. Additionally, we will evaluate military burn care education, focusing on self-care, comrade aid, and alignment with civilian training.

**Results:** Registration will enable quantification of incidence, injury patterns, infection rates, and outcomes, and allow comparison with civilian cohorts. AI tools will support real-time triage, burn assessment, and decision-making. Educational strategies will strengthen self-aid and buddy care competencies and improve alignment between military and civilian training.

**Conclusions:** This integrated program combines data registration, AI-supported decision-making, and targeted education to address key gaps in military burn care. By improving early assessment and frontline interventions, it supports timely care from injury to rehabilitation and provides a scalable framework to enhance preparedness and outcomes in conflict and disaster settings. Importantly, these innovations are directly translatable to civilian burn care, contributing to improved triage, treatment, and education in resource-limited and emergency settings.

**Keywords:** military; education; trauma

### 5.7. P10 a Single Burn Center Response to a Mass Casualty Incident: Surgical Strategy and Outcomes

**Jussi Valtonen** ^1^, Dr. Simon Myers, Dr. Hischam Taha, Dr. Islam Abdulrahman, and Ms. Deborah Pierce

1. Sheikh Shakhbout Medical City, Abu Dhabi, United Arab Emirates

**Introduction:** Burn injuries from mass casualty incidents vary in severity, total body surface area (TBSA) involvement, and anatomical distribution, influencing surgical planning, operative time, and hospital stay.

**Methods:** We retrospectively reviewed 22 patients (age 20–42 years; 21 males, 1 female) admitted to a single burn center between 25th January and 29th January 2026 following a gas explosion. The center operated with four burn surgeons across two shifts; the average daily census across the dedicated burn service was 28.9 (Jan.) and 24.9 (Feb.). The center maintained normal acute burn care throughout, without referring or denying other patients. Data included demographics, TBSA, burn classification, inhalation injury, excised percentage, anatomical distribution, timing to first surgery, operative time, ICU and hospital length of stay (LOS), surgical approach, and modified Baux score.

**Results:** Burns ranged from 13 to 53% TBSA, predominantly second-/third-degree; inhalation injury was present in 11 patients. The upper/lower limbs, head, and trunk were most affected. Twenty patients underwent excision and grafting; one patient died before surgery, and one required no surgery. Average time from admission to first surgery was 24 h. Surgical strategy used one- or two-step approaches: smaller burns were excised with immediate autografts; larger burns involved staged excision with dermal substitute/allograft followed by autograft coverage. All patients achieved full coverage within two major operations; additional procedures addressed minor patch-ups or facial contractures. Total surgeries averaged 1.6 per patient; cumulative operative time ranged between 22 and 200 min, averaging 3.1 min/%TBSA. LOS per % TBSA averaged 1.4 days/%TBSA, ICU LOS 0–18 days, and total LOS 19–74 days. Modified Baux scores ranged from 33 to 95, with an average of 58. Excellent outcomes were achieved through strong institutional support and a highly engaged, collaborative surgical team.

**Conclusions:** Burn severity, TBSA, and anatomical distribution strongly predict operative burden and LOS. Maintaining normal acute service alongside mass casualty care, with early staged surgical planning, enables complete coverage within two operations and optimizes outcomes.

### 5.8. P11 Carbon Monoxide Poisoning and the Use of Hyperbaric Oxygen Therapy in Mass Casualty Incidents

**Dr. Radovan Čelóvský** ^1^, Peter Lengyel ^1^, Erik Eliáš ^1^, Dana Kolesár ^1^, and Jana Horváthová ^1^

1. Department of Burns and Reconstructive Plastic Surgery, Faculty Hospital Agel Košice-šaca, Košice, Slovakia

**Aim:** To describe current guidelines, triage strategies, and the use of hyperbaric oxygen therapy (HBOT) in patients with carbon monoxide poisoning during mass casualty incidents.

**Methods:** This study reviews current guidelines for the management of carbon monoxide poisoning, including triage principles, carboxyhemoglobin levels, and neurological assessment. Additionally, we present a 20-year retrospective analysis of 9553 patients treated at our institution, focusing on the indications and outcomes of hyperbaric oxygen therapy.

**Results:** Duration of exposure, elevated carboxyhemoglobin levels, and the presence of neurological symptoms were identified as key factors contributing to tissue hypoxia severity.

**Conclusions:** Carboxyhemoglobin levels and neurological assessment are critical for optimizing patient triage and minimizing the adverse outcomes of carbon monoxide poisoning. Early and appropriate use of hyperbaric oxygen therapy may improve clinical outcomes, particularly in mass casualty settings.

**Keywords:** carbon monoxide poisoning; hyperbaric oxygen therapy; mass casualty incidents

### 5.9. P14 a Multidisciplinary Rehabilitation Approach for Burn Survivors of the 2025 Kochani Fire Disaster

**Md. Katarina Mirchovska** ^1^ and Ass. Gordana Georgieva

1. General Hospital Kochani, Kochani, North Macedonia

**Introduction and Background:** In June 2025, the City of Kochani experienced a devastating fire disaster, resulting in 193 injured young individuals suffering from complex burns and inhalation trauma. To address the long-term physical and psychological sequelae, the Macedonian Government and General Hospital Kochani organized a comprehensive rehabilitation camp at Ponikva.

**Design and Methodology:** The program, running from 18 June to 27 August 2025, utilized a multidisciplinary approach designed to bridge the gap between clinical stabilization and social reintegration. The clinical interventions included: physical therapy with specialized scar massage techniques that prevent contractures and improve skin elasticity; group exercises and sports to rebuild cardiovascular endurance and muscular strength, ideal for improving respiratory function; psychological support—both individual counseling and group therapy sessions to address Post-Traumatic Stress Disorder and body image concerns, and social reintegration—the camp integrated social and creative activities to foster community and resilience through painting, puzzles, cinema nights, and weekends featuring live bands and music therapy.

**Results and Conclusions:** The Ponikva camp proved highly productive and transformative for the participants. By the conclusion of the summer program—six months post-tragedy—the majority of patients reported significant improvements in mobility, respiratory ease, and mental health. The holistic nature of the camp empowered survivors to successfully return to their daily activities. This initiative serves as a model for government-led disaster response, highlighting the necessity of combining medical physiotherapy with environmental therapy and psychological social support systems.

**Keywords:** Ponikva camp; Kochani fire

### 5.10. P15 Impact of Rehabilitation Intensity During Usual Operations Versus Mass Casualty Surge Periods on Burn Patient Outcomes: A Comparative Analysis

Deborah Pierce ^1^, **Katharina Fischbach Reinhardt** ^1^, and Reem Beck ^1^

1. Sheikh Shakhbout Medical City, Abu Dhabi, United Arab Emirates

**Aim:** To compare rehabilitation intensity and functional outcomes in adults with severe burns (>20% TBSA) during usual operational conditions versus mass casualty surge periods.

**Methods:** A retrospective comparative study was conducted in a tertiary burn center rehabilitation unit. Adult patients with burns exceeding 20% TBSA were analyzed across two operational contexts: usual conditions and mass casualty surge periods marked by increased patient volume, higher burn severity, and constrained resources. Rehabilitation intensity was measured using therapy minutes per cutaneous functional unit (CFU) and session frequency, comparing prescribed versus delivered care. Outcomes assessed included AM-PAC mobility scores, documented reduced range of motion at discharge, and length of stay (LOS) normalized to CFU and TBSA.

**Results:** During surge conditions, delivered rehabilitation intensity decreased from 100% to 62% of prescribed levels, with therapy minutes falling from 2.0 to 1.2 min/CFU/day and sessions from 1.3 to 0.8 per CFU. Despite reduced intensity, outcomes were comparable or improved. The surge group demonstrated a faster rate of AM-PAC mobility improvement (1.12 vs. 0.9). Rates of reduced range of motion at discharge were similar (41% surge vs. 39% usual). LOS/CFU was reduced during surge periods (0.5 vs. 0.6 days/CFU).

**Conclusions:** Although rehabilitation delivery was reduced during mass casualty surges, adaptive prioritization strategies maintained core functional outcomes. These findings highlight the flexibility and resilience of rehabilitation services during capacity-surpassing events.

**Keywords:** burn rehabilitation; intensity; mass casualty

### 5.11. P16 Interactive Impact of Blue Light, Cold Argon Plasma and Amino Acids in the Treatment of Deep Dermal and Full-Thickness Burns

**Panche Taskov** ^1,2^, Zorin Crainiceanu, Daniela Taskov, Gaurav Narad, Viviana Narad, Eniko Hordovan, Ancuta Pop Coman, Andreea Babii, Robert Blendea, Vlad Dragunescu, Daniela Corodati, and Wael El Amine

1. University of Medicine and Pharmacy “Victor Babes”, Timisoara Romania, Timisoara, Romania
2. Plastic and Reconstructive Surgery Department Casa Austria, County Emergency Clinical Hospital “Pius Branzeu”, Timisoara Romania, Timisoara, Romania

**Introduction:** Tangential excision and split-thickness grafting remain the reference for deep burns but are blood-intensive, donor-site-limited and frequently yield hypertrophic scars.

We evaluated a multimodal regenerative protocol coupling selective EDNX with autologous SMG, PRP, MN, CAP, BL, HA and AA topics.

**Methods:** A single-center retrospective study (2025) compared consecutive adults with grade IIB/III burns treated with PAN-EDNX with size and depth-matched SOC controls.

Primary endpoints were time to complete eschar removal, need for secondary surgery, and safety of the treatment.

**Results:** Baseline age (35.3 ± 11.2 y vs. 43.1 ± 7.8 y) and %TBSA (36.3 ± 4.3 vs. 33.2 ± 4.3) were comparable; ABSI scores did not differ.

PAN-EDNX halved eschar clearance time (5.0 ± 1.1 vs. 9.1 ± 1.5 d) and eliminated secondary grafting (0% vs. 93%).

Spontaneous/stimulated epithelialization occurred in every PAN-EDNX case vs. 7% of SOC cases.

No procedure-related bleeding, electrolyte disorders or mortality differences were observed.

**Conclusions:** The PAN-EDNX concept accelerates eschar clearance, obviates grafting, improves scar quality and shortens rehabilitation without compromising safety.

Dermis preservation and micro-graft bio-stimulation appear synergistic, supporting wider integration of regenerative adjuncts into enzymatic debridement pathways for extensive burns.

The clinical results obtained via therapeutic flowchart based on “PAN-EDNX” demonstrate excellent esthetic outcomes as well as functionality restoration.

This novel regenerative technique has shown promising results in the burn healing process, reduced costs and hospitalization, decreased lost working days and increased quality of life.

“PAN-EDNX” is a safe procedure and aids in the real challenge of personalized care in burn treatment.

### 5.12. P17 from Evidence to Practice: Designing a Histopathological Assessment Protocol for Burns After NexoBrid^®^ Enzymatic Debridement

**MD. Stefana Avadanei-Luca** ^1,2^, MD. Andra-Irina Bulgaru-Iliescu ^1,2^ and Prof. Mihaela Pertea ^1,2^

1. Grigore T Popa University of Medicine and Pharmacy/Sf Spiridon Burn Unit, Iasi, Romania
2. Sf Spiridon Emergency County Hospital Burn Unit Iasi Romania, Iasi, Romania

**Introduction:** To design a standardized, evidence-based histopathological sampling and evaluation protocol for burn wounds treated with anacaulase-bcdb (NexoBrid^®^), translating the available preclinical and clinical evidence into a reproducible clinical and research tool.

**Methods:** The protocol was developed through critical appraisal and synthesis of the existing preclinical and clinical histopathological literature on bromelain-based enzymatic debridement, validated burn biopsy methodology, established viability scoring systems, and the product’s regulatory guidance. Four sampling timepoints were defined: pre-debridement baseline (T0), immediate post-debridement (T1), depth progression biopsy at 24–48 h (T2), and pseudo-eschar work-up at day 6–10 (T3), with standardized site selection rules targeting the deepest avascular zone and the transitional wound bed interface. A core staining panel—H and E, LDH viability histochemistry, and Verhoeff–Van Gieson elastic stain—was specified alongside a structured quantitative report template incorporating microvascular patency depth measurement and a 0–4 H and E viability score.

**Results:** The principal histological signals described in the literature—upper dermal homogenisation, vascular congestion, deep dermal preservation, and transepithelial elastic fiber elimination in pseudo-eschar—are operationalized into reproducible, clinically actionable biopsy and reporting standards, with inter-rater calibration targets and defined turnaround times for each analytical pathway.

**Conclusions:** A practical framework for prospective histopathological data generation following NexoBrid^®^ debridement is provided, supporting informed graft-versus-conservative decisions and pseudo-eschar characterization. Multicenter adoption is required to validate scoring thresholds and address the critically limited human histology dataset currently available.

**Keywords:** enzymatic debridement; burn wound histopathology; anacaulase-bcdb

### 5.13. P18 the VISTA Score (Viability-Integrated Staged Tissue Assessment): A Structured Framework for Standardizing Debridement Decisions in Mixed-Depth Burns

**Dr. Dan Cristian Moraru** ^1^, Andra Irina Bulgaru ^1,2^, Stefana Avadanei-Luca ^1,2^, Codrin Constantinescu ^1,2^, Alexandru Hristo Amarandei ^1,2^, Angelica Balaur ^2^, and Mihaela Pertea ^1,2^

1. “Grigore T. Popa” University of Medicine and Pharmacy, Iasi, Romania
2. Burn Unit, “Sf. Spiridon” Emergency Hospital, Iasi, Romania

**Aim:** To develop and implement the VISTA score (Viability-Integrated Staged Tissue Assessment), a structured multimodal decision framework tool designed to objectively standardize intraoperative decision-making during sequential debridement of mixed-depth burns, improving reproducibility of surgical judgment and supporting precision-oriented burn care.

**Methods:** The VISTA framework was developed through structured analysis of intraoperative decision-making challenges in a regional burn unit and applied to a consecutive series of adult patients presenting with mixed-depth thermal injuries. The scoring system integrates three independent viability domains: (1) tissue perfusion, assessed through clinical indicators and adjunctive perfusion imaging; (2) dermal structural integrity, evaluated by consistency, color, and bleeding pattern; (3) dynamic wound evolution, assessed through serial demarcation across staged procedures. Each domain is scored 0–2 points, generating a cumulative index of 0–6. Predefined thresholds guide surgical action: continued excision (0–2), temporary cessation with reassessment (3–4), or tissue preservation with delayed reconstruction (5–6).

**Results:** Application of the VISTA framework provided a consistent operational structure for intraoperative decision-making and improved consistency of excision endpoints across operators. More selective tissue management strategies were observed, with stable infection control, graft take, and wound closure outcomes during early follow-up. Formal prospective validation with inter-rater reliability assessment is in preparation.

**Conclusions:** The VISTA score introduces a reproducible, threshold-based method for guiding precision debridement in mixed-depth burns, supporting the transition toward algorithm-driven burn surgery and providing a foundation for future multicenter validation.

**Keywords:** burns; debridement; tissue viability

### 5.14. P20 the New Multimodal Approach in the Treatment of Deep Dermal and Full-Thickness Facial Burns—Psychological Impact and Social Reintegration

**Panche Taskov** ^1^, Daniela Taskov, Zorin Crainiceanu, Gaurav Narad, Viviana Narad, Eniko Hordovan, Ancuta Pop Coman, Andreea Babii, Robert Blendea, Vlad Dragunescu, Daniela Corodati, and Wael El Amine

1. University of Medicine and Pharmacy “Victor Babes”, Timisoara Romania, Timisoara, Romania
2. Plastic and Reconstructive Surgery Department Casa Austria, County Emergency Clinical Hospital “Pius Branzeu”, Timisoara Romania, Timisoara, Romania

**Introduction:** Deep facial burns are often a challenge, especially due to the fact that they are often associated with respiratory tract burns that lead to the rapid destabilization of the patient.

The preservation of viable facial layers, especially the viable dermis, is essential for spontaneous epithelization.

Carrying out the affected tissue debridement as quickly as possible is essential, especially in the case of burns on the face, where early debridement promises a better esthetic and functional result.

**Methods:** A single-center retrospective observational study was performed over a 5-year period (2020–2025), comprising 15 adults with deep partial/full-thickness facial burns treated with EDNX, three of them with airways burns, with an score of ABSI of 7. The facial cohort showed a mean age of 35.6 ± 10.8 years, average% TBSA was 23.8 ± 7.0, and with 6 ± 2% TBSA of the face treated by EDNX.

**Results:** All patients with deep partial/full-thickness facial burns achieved full epithelialization within three weeks, reflecting the high vascularity of facial skin synergized with dermis-sparing debridement.

Functional assessment revealed preserved eyelid excursion, oral competence and cervical extension, a notable advance over traditional excision outcomes that often necessitate later scar-release procedures.

**Conclusions:** Off-label facial application EDNX is safe and delivers a significantly shorter length of stay while preserving dermal tissue that translates into superior long-term scar quality and a clinically advantageous alternative to early tangential excision. Facial use eliminates grafting, accelerates healing and yields near-normal scarring, with high patient satisfaction, intact facial functioning, and faster occupational reintegration and increased quality of life.

**Keywords:** EDNX; facial burns

### 5.15. P21 Reconstruction of Complex Wounds with Exposed Bone Following Necrotizing Fasciitis Using a Biodegradable Temporizing Matrix

**Dr. Julie van Durme** ^1^, Ms. Kim De Mey, Mr. Jozef Verbelen, Ms. Tine Nuyttens, Ms. Petra De Coninck and Prof. Dr. Karel Claes

1. Burn Center University Hospital Ghent, Belgium, Ghent, Belgium

**Introduction:** Necrotizing fasciitis is a rapidly progressive soft tissue infection characterized by widespread fascial necrosis and high morbidity. Prompt surgical debridement combined with broad spectrum antibiotics is essential for survival, often resulting in extensive soft tissue defects. Reconstruction of these defects is particularly challenging, and even more so when critical structures such as bone are exposed. Dermal substitutes, including Novosorb biodegradable temporizing matrices (BTM), have emerged as promising tools in such complex cases.

**Methods:** We report a single-center case report of a 68-year-old male with necrotizing fasciitis of the left lower limb. Following multiple radical debridements, Novosorb BTM was applied to the anterior side of the left lower limb with exposed tibia, according to a standardized protocol. After sufficient neovascularization, the sealing membrane was removed and the neodermis was covered with split-thickness skin grafts (STSGs). Clinical data including patient characteristics, wound etiology and location, treatment timeline, healing outcomes, complications, and hypertrophic scar formation were collected.

**Results**: BTM was applied five weeks after initial presentation on the anterior side of the left lower limb. The sealing membrane was removed after eight weeks, followed by STSG coverage. Complete wound closure was achieved within ten days post-grafting. The patient demonstrated favorable functional and esthetic outcomes.

**Conclusions**: BTM represents an effective reconstructive option for complex wounds with exposed bone following necrotizing fasciitis. It facilitates durable wound coverage, promotes satisfactory cosmetic results, and may reduce surgical burden in medically complex patients.

**Keywords:** burn; biodegradable temporizing matrix; synthetic dermal substitute

### 5.16. P22 Necrosectomy with Nanocrystalline Silver Dressings Versus Autografting in Deep Dermal Burns: A Case-Based Analysis Supported by Laser Doppler Imaging

Dr. Marina Dobreva-Petkova^1^, Velina Zdravkova^1^, Dimitar Dimitrov^1^, and **Martin Popov** ^1^

1. Umbalsm “n.i. Pirogov” Sofia, Bulgaria, Sofiq, Bulgaria

**Objectives:** To compare outcomes between necrosectomy followed by nanocrystalline silver dressing and split-thickness skin grafting (STSG) in deep dermal burns, utilizing laser doppler imaging (LDI) to support individualized, tissue-preserving clinical decisions.

**Methods:** Three representative clinical cases of deep dermal (IIB) burns were analyzed: one intra-patient comparison of symmetrical burn areas treated with both approaches, one limited total body surface area (TBSA) burn with LDI-guided assessment, and one extensive burn utilizing selective grafting. LDI was performed at 48–72 h post-injury to evaluate tissue perfusion. Assessed outcomes included time to epithelialization, need for surgical intervention, clinical scar quality, and functional outcome.

**Results:** LDI assessment enabled the identification of areas with intermediate perfusion suitable for conservative management. Epithelialization in areas treated with nanocrystalline silver dressings occurred within 15–24 days, comparable to grafted regions. In the intra-patient comparison, non-grafted areas demonstrated similar clinical and esthetic outcomes without donor-site morbidity. In extensive burns, the selective grafting strategy reduced the operative burden without compromising healing.

**Conclusions:** Necrosectomy followed by nanocrystalline silver dressing represents a reliable alternative to autografting in selected deep dermal burns, particularly when supported by LDI-based assessment. These findings support a more individualized, tissue-preserving surgical approach that significantly reduces surgical burden and minimizes donor-site morbidity.

**Keywords:** deep dermal burns; nanocrystalline silver; laser doppler imaging

### 5.17. P23 Standard of Care in Burn Wound Management: A Case Report Following the Crans-Montana Incident

**Kim De Mey** ^1^, Julie Van Durme ^1^, Jozef Verbelen ^1^, Petra De Coninck ^1^, Tine Nuyttens ^1^, and Karel E.Y. Claes ^1^

1. UZ Gent, Ghent, Belgium

**Background:** A fire at a bar in Crans-Montana, Switzerland, resulted in 41 deaths and over 100 injuries, involving severe burns. One patient was transferred to our Burn Center at Ghent University Hospital. At our center, an enzyme alginogel (Flaminal^®^) is the standard of care for conservative burn treatment. This study evaluates its efficacy in intermediate-depth burns and its use following enzymatic debridement with NexoBrid^®^ (EDNX) in deeper burns.

**Methods:** We report a single-center case of a 17-year-old patient with intermediate-depth and deep burns. Data collected included burn characteristics, treatment strategies, bacteriology, time to wound healing, length of stay (LOS) and scar management.

**Results:** The patient sustained a 15.5% TBSA flame burn with grade 2–3 inhalation injury. Burns involved four regions of interest (ROIs): intermediate-depth burns on the lower back (4%), and deeper burns on the face (4%) and hands (7.5%). Fasciotomies and escharotomies were performed on day 1. EDNX was performed on the face and hands on day 3. Flaminal^®^ was used on the lower back and hands, while Furacine^®^ was initiated for a facial infection. No infections occurred in the Flaminal^®^-treated areas. Split-thickness skin were applied to both hands 26 days after EDNX. The patient was hospitalized for 35 days before transfer for rehabilitation.

**Conclusions:** Flaminal^®^ is an effective dressing for conservative burn management and post-EDNX care. Its autolytic debridement capacity, absorptive properties, and antimicrobial enzyme system (GLG) contribute to improved wound healing and infection prevention, resulting in favorable functional and esthetic outcomes.

**Keywords:** intermediate-depth burns; deep burns; SOC; Flaminal^®^; Crans-Montana

### 5.18. P24 Clinical Outcomes of Acellular Fish Dermis in Hostile Open Abdomen Management: A Multicenter Experience

**Alfredo Cordova** ^1,2^, Carrie McGroarty ^3^, Kristy Miller ^1^, Talia Selembo ^1^, Scott Hultman ^3^

1. Sarasota Memorial Hospital, Sarasota, United States, ^2^Burn Center, Temple University Hospital, Philadelphia, United States, ^3^WakeMed Health & Hospital, Raleigh, United States

**Introduction:** Managing an open abdomen is a surgical challenge, frequently complicated by high morbidity and risks such as entero-cutaneous fistulae. While biologic and synthetic skin substitutes offer temporary coverage, decellularized and lyophilized fish dermis has emerged as a promising alternative for promoting a vascularized wound bed. This multicenter case series evaluates the efficacy of fish dermis in managing complex abdominal catastrophes.

**Methods:** Ten-patients at 3-diferent institutions presenting with life-threatening conditions—including septic shock, acute peritonitis, and abdominal catastrophes with loss of domain—underwent wound bed preparation using acellular fish dermis. Etiologies included necrotizing soft tissue infections and abdominal compartment syndrome, often exacerbated by comorbidities like diabetes and renal insufficiency. Following stabilization through debridement and decompressive laparotomy, exposed bowel was resurfaced with cod fish skin xenografts. Upon establishment of a viable granulation bed, definitive closure was achieved using autologous split-thickness skin grafts (STSG).

**Results:** The fish dermis integrated rapidly; optimal granulation tissue covered over 95% of the wound surface within 14 days, with some cases showing integration as early as 5 days post-application. This accelerated integration provided a robust foundation for secondary grafting. Subsequent meshed STSG achieved nearly 100% graft take across all cases within a two-week period.

**Conclusions:** Decellularized fish dermis effectively prepares complex open abdominal wounds for definitive autografting by promoting rapid, high-quality granulation. These findings suggest that fish skin xenografts are a viable option for enhancing autograft success in challenging clinical scenarios. Further large-scale, multicenter studies are warranted to validate these results.

**Key Words**: fish dermis, open abdomen, enterocutaneous fistula.

### 5.19. P25 Polylactic Acid-Based Dermal Matrix Enhances Autologous Graft Take in Complex Gunshot Blast Injuries: A Case Series

**Alfredo Cordova** ^1,2^, Chloe Maugans ^1^, Kristy Miller ^1^, Talia Selembo ^1^

1. Sarasota Memorial Hospital, Sarasota, United States,
2. Burn Center, Temple University Hospital, Philadelphia, United States

**Introduction:** Blast injuries from gunshot wounds (GSWs) present significant reconstructive challenges due to extensive tissue loss, compromised vascularity, and high infection risk. For severe defects, advanced surgical interventions—such as the use of dermal substitutes—are often required. This study evaluates the efficacy of a polylactic acid (PLA) skin substitute in promoting wound bed vascularization and infection control in complex traumatic wounds.

**Methods:** We report on two cases of complex GSWs (one accidental, one self-inflicted). Management involved serial debridement and irrigation, followed by the application of a PLA-based dermal matrix to optimize the wound bed for definitive reconstruction. Negative pressure wound therapy (NPWT) was utilized concomitantly. Following the development of a viable granular bed, autologous skin grafting was performed.

**Results:** In both cases, the PLA matrix facilitated the formation of a highly vascularized granulation bed, with of the wound surface area ready for grafting within 14 to 21 days. Subsequent reconstruction with meshed split-thickness skin grafts (STSGs) achieved nearly 100% graft take. Complete epithelialization was observed within 10 days of the secondary grafting procedure. No matrix-related complications or infections were noted.

**Conclusions:** A polylactic acid copolymer-based epidermal skin substitute appears to be a safe and effective adjunct in the management of complex blast injuries. By accelerating the formation of a robust, vascularized wound bed, this matrix may expedite the definitive closure of traumatic wounds. While these results are promising, larger prospective cohort studies are warranted to validate these findings and standardize treatment protocols.

**Keywords:** polylactic acid, dermal matrix, blast injury, gunshot wound

### 5.20. P26 the Role of Hyperbaric Oxygen Therapy in Burns—A Case Series from a Single-Center Experience

**Elias Zekri** ^1,2^, Dr, MD Tomaz Oliveira ^3^, Dr, MD Carla Amaro ^1,4,5^ and Dr, MD, PhD José Guimarães-Ferreira ^1,3^

1. Lisbon School of Medicine, Lisbon, Portugal
2. Portuguese Naval Academy, Lisbon, Portugal
3. Plastic Surgery Deparment of Centro Hospitalar Universitário Lisboa Norte, Lisbon, Portugal
4. Estado-Maior General das Forças Armadas, Lisbon, Portugal
5. Otorhinolaryngology Unit of Hospital CUF Descobertas, Lisbon, Portugal

**Objectives:** Burn injuries represent a significant global health burden, resulting in a statistically significant burn-related death rate. Despite the existence of standardized treatment protocols, major challenges persist regarding patients’ susceptibility to burn-related complications and prolonged hospitalization. Hyperbaric oxygen therapy (HBOT) has recently gained attention for its role in tissue regeneration, with recent European consensus statements reinforcing its therapeutic value in several clinical conditions, especially burn management. This case series aims to provide practical insights into the therapeutic potential of HBOT for optimizing outcomes in burn wound care.

**Methods:** A retrospective case series of seven patients treated at the Hyperbaric and Subaquatic Medicine Center (CMSH—Hospital das Forças Armadas, Polo, Lisboa) was conducted. Following institutional ethical and scientific approval, digital and physical medical records indexed under “Burn Wounds” were reviewed. Exclusion criteria comprised patients with insufficient clinical data and those who failed to initiate HBOT following their initial eligibility consultation.

**Results:** These cases provide practical insight into the integration of HBOT in acute and chronic burn wound management protocols and local infection control. The evidence points to potential improvements in wound healing and reductions in complication and hospitalization rates, though challenges remain in uniformly implementing HBOT strategies and dealing with logistical incompatibilities between institutions.

**Conclusions:** This case series emphasizes the continuing challenges of burn wound management, exploring the application of hyperbaric oxygen and its potential benefit in enhancing clinical outcomes of the burnt patient. Further investigation is appropriate for the uniformization of treatment protocols and patient selection.

**Keywords:** burn wounds; hyperbaric oxygen therapy; clinical outcomes

### 5.21. P50 the Wound Behind the Wound: Suicide-Related Burns in a Tertiary Burn Unit, a 14-Year Experience

**Ana Beatriz Garrido**, José Manuel Azevedo, Inês Catalâo, Miguel Sitima, Rui Calvinho Almeida, Dr. Marta Carvalhas de Almeida, Sara Ramos, Susana Pinheiro, Luis Cabral

**Background:** Self-inflicted burns represent a uniquely severe and complex subset of burn injuries, combining a surgical and psychiatric emergency simultaneously. Despite their clinical severity, this population remains poorly characterized in the literature and formal psychiatric care pathways in burn units are largely absent. 

**Objectives:** To characterize suicide-related burn admissions in a single tertiary burn unit, comparing their demographic, clinical and psychiatric profile with the general burn population. 

**Methods:** Retrospective study of all admissions to the Burn Unit of Coimbra between January 2012 and April 2026. Suicide-related burns were identified and compared with the remaining 1991 admissions regarding demographics, burn severity, mechanism and outcomes. Psychiatric profile was additionally characterized for the suicide group including prior diagnosis, previous attempts, follow-up at time of burn and substance abuse. 

**Results:** Among 2023 admissions, 32 (1.58%) were suicide related. Compared to the general burn population, suicide-related burns affected younger patients (mean age 52.0 vs 59.2 years) and were predominantly male (65.6% vs. 56.4%). Flame and fire were the overwhelming mechanism in the suicide group (84.4%), whereas the general population showed a more diverse distribution including a substantial proportion of scald injuries (28.0%). Suicide-related burns were significantly more severe, with a mean TBSA of 30.8% versus 19.6%, full thickness burns in 81.2% versus 43.6%, and a mean length of stay of 42.6 versus 21.4 days. Mortality in the suicide group reached 28.1% compared to 9.5% in the general population. Inhalation injury was present in 50.0% of cases. Of the 32 patients, 81.2% had a prior psychiatric diagnosis, 32.3% had a documented previous suicide attempt, 50.0% were under psychiatric follow-up at the time of the burn and 28.1% had a history of substance abuse including alcohol and illicit drugs. While 73.9% of survivors were discharged with psychiatric follow-up, the majority returned to pre-existing care pathways rather than receiving formal reassessment or intensification of psychiatric support following their attempt. Notably, one patient was readmitted within one month with a second self-immolation burn. 

**Conclusions:** Suicide-related burns carry a mortality nearly three times higher than accidental burns, with significantly larger and deeper injuries and longer hospitalization. Despite the majority carrying a prior psychiatric diagnosis, half had no active follow-up at the time of their burn and most survivors were discharged back to pre-existing care without formal reassessment. These findings highlight an opportunity for burn units to play an active role in identifying vulnerable patients and ensuring robust psychiatric and addiction support pathways at discharge. 

### 5.22. P51 Selective Enzymatic Debridement of Hand Burns with Nexobrid®: Surgical Avoidance and Clinical Outcomes in an Eight-Patient Series 

**Marta Carvalhas de Almeida**, José Miguel Azevedo, Inês Catalão, Miguel Sitima, Rui Almeida, Ana Beatriz Garrido, Miguel Vaz, Carla Diogo, Susana Pinheiro, Luis Cabral

**Aim:** To evaluate the effectiveness of selective enzymatic debridement with Nexobrid® in acute hand burns, focusing on surgical avoidance and short-term clinical and functional outcomes.

**Methods:** A retrospective analysis was conducted of eight patients treated with Nexobrid® for burns involving at least one hand at Coimbra Burns Unit, Portugal, between 2022 and 2026. Demographic data, burn characteristics, timing of application, analgesia modality, need for complementary surgical debridement or grafting, complications, length of stay and early functional outcomes were analysed. Mean age was 44 ± 21 years, and mean burn extent was 12.0 ± 11.7% TBSA. Nexobrid® was applied between 24–48 hours post-injury in all cases, with a 4-hour contact time. All patients received advanced analgesia, including regional nerve blocks or sedoanalgesia, according to clinical indication. 

**Results:** 6/8 patients (75%) required no additional surgical debridement in the areas treated with Nexobrid®. 2/8 patients (25%) required tangential excision and split-thickness skin grafting. No significant bleeding, graft loss or procedure-related adverse events occurred. Early mobilisation was possible in all cases, with good functional recovery at 1 month in all evaluated cases. No functional contractures were observed at short-term follow-up. 

**Conclusions:** Nexobrid® was effective and safe for selective debridement of hand burns, enabling surgical avoidance in most cases and supporting early functional recovery. These findings reinforce its value as a first-line debridement strategy in functionally critical areas, particularly in patients where surgical morbidity is a concern. Future research should assess long-term functional and scar-related outcomes after enzymatic debridement of hand burns. 

